# Supplementary material for: Influence of Early Life Factors on the Breast Milk and Fecal Microbiota of Mother–Newborn Dyads
Source: Microorganisms. 2024 Oct 25;12(11):2142. doi: 10.3390/microorganisms12112142 (PMC11596411; doi:10.3390/microorganisms12112142)
Supplement: Supplementary file 1 [file microorganisms-12-02142-s001.zip › microorganisms-3123245-SI.pdf]

Supplementary Material for

Influence of early life factors on the breast milk and fecal microbiota of mother-newborn dyads

Emmanuel Cervantes-Monroy, Imelda C. Zarzoza-Mendoza, Samuel Canizales-Quinteros, Sofia Moran-Ramos, Judith Villa-Morales, Blanca López-Contreras, Fairt V. Carmona-Sierra [5], and Maricela Rodríguez-Cruz

Corresponding author: Email: maricela.rodriguez.cruz@gmail.com

The Supplementary Material includes.

1. **Results**
    - **1.1. Supplementary Tables**
        - *1.1. Supplementary Table 1*
        - *1.2. Supplementary Table 2*
        - *1.3. Supplementary Table 3*
        - *1.4. Supplementary Table 4*
        - *1.5. Supplementary Table 5*
        - *1.6. Supplementary Table 6*

1. Supplementary Tables

Supplementary Table S1. Maternal gut microbiota composition at phylum level.

Phylum	Number of genera	Mean relative abundance (%)
Firmicutes	181	60.2665
Bacteroidota	26	31.3971
Actinobacteriota	31	3.9583
Proteobacteria	44	2.6712
Verrucomicrobiota	7	1.0345
Desulfobacterota	4	0.2800
Euryarchaeota	1	0.2095
Cyanobacteria	1	0.0989
Elusimicrobiota	1	0.0432
Fusobacteriota	3	0.0230
Unclassified	1	0.0122
Campilobacterota	1	0.0039
Thermoplasmatota	1	0.0011
Synergistota	1	0.0005
Bdellovibrionota	1	0.0002
**Total**	**304**	**100**

Supplementary Table S2. Maternal gut microbiota composition at genus level stratified by phylum.

Genus	Mean relative abundance (%)
Firmicutes	**60.27**
Other Firmicutes	41.88
Blautia	6.05
Faecalibacterium	5.41
Oscillospiraceae UCG-002	2.63
Christensenellaceae R-7	2.35
Subdoligranulum	1.95
Bacteroidota	**31.40**
Bacteroides	17.12
Prevotella	9.27
Alistipes	1.69
Other Bacteroidota	1.32
Parabacteroides	1.15
Paraprevotella	0.85
Actinobacteriota	**3.96**
Bifidobacterium	3.28
Collinsella	0.48
Other Actinobacteriota	0.09
Eggerthella	0.05
Gordonibacter	0.03
Adlercreutzia	0.02
Proteobacteria	**2.67**
Other Proteobacteria	1.18
Sutterella	0.70
Escherichia-Shigella	0.58
Parasutterella	0.16
Pseudomonas	0.05
Succinivibrio	0.01
Others	**1.71**
Other taxa	1.71
**Total**	**100**

Supplementary Table S3. Breast milk microbiota composition at phylum level.

Phylum	Number of genera	Mean relative abundance (%)
Firmicutes	157	59.1724
Proteobacteria	108	27.1315
Actinobacteriota	42	7.0116
Bacteroidota	45	5.7910
Unclassified	1	0.2938
Fusobacteriota	4	0.1665
Verrucomicrobiota	7	0.1371
Planctomycetota	6	0.1171
Acidobacteriota	8	0.0704
Desulfobacterota	4	0.0324
Myxococcota	4	0.0197
Spirochaetota	1	0.0146
Cyanobacteria	2	0.0110
Bdellovibrionota	1	0.0075
Euryarchaeota	1	0.0060
Synergistota	1	0.0051
Patescibacteria	3	0.0034
Elusimicrobiota	1	0.0028
WPS-2	1	0.0026
Campilobacterota	2	0.0023
Chloroflexi	1	0.0006
Gemmatimonadota	1	0.0003
Fibrobacterota	1	0.0003
**Total**	**402**	**100**

Supplementary Table S4. Breast milk microbiota composition at genus level stratified by phylum.

Genus	Mean relative abundance (%)
Firmicutes	**59.17**
Streptococcus	31.52
Staphylococcus	17.46
Other Firmicutes	6.77
Gemella	1.91
Faecalibacterium	0.87
Blautia	0.64
Proteobacteria	**27.13**
Other Proteobacteria	14.95
Pseudomonas	4.90
Stenotrophomonas	2.55
Herbaspirillum	2.09
Escherichia-Shigella	1.40
Acinetobacter	1.25
Actinobacteriota	**7.01**
Bifidobacterium	4.05
Corynebacterium	2.22
Rothia	0.48
Other Actinobacteriota	0.12
Actinomyces	0.11
Collinsella	0.02
Bacteroidota	**5.79**
Bacteroides	2.07
Prevotella	1.83
Other Bacteroidota	1.04
Alistipes	0.49
Nubsella	0.20
Sphingobacterium	0.17
Others	**0.89**
Other taxa	0.89
**Total**	**100**

Supplementary Table S5. Newborn gut microbiota composition at phylum level.

Phylum	Number of genera	Mean relative abundance (%)
Proteobacteria	41	35.5038
Actinobacteriota	23	30.4233
Firmicutes	113	24.0218
Bacteroidota	17	10.0156
Verrucomicrobiota	1	0.0222
Desulfobacterota	4	0.0051
Unclassified	1	0.0050
Campilobacterota	1	0.0008
Elusimicrobiota	1	0.0006
Fusobacteriota	1	0.0005
Synergistota	1	0.0005
Euryarchaeota	1	0.0004
Cyanobacteria	1	0.0001
Bdellovibrionota	1	0.0001
**Total**	**402**	**100**

Supplementary Table S6. Newborn gut microbiota composition at genus level stratified by phylum.

Genus	Mean relative abundance (%)
Proteobacteria	**35.50**
Other Proteobacteria	18.26
Escherichia-Shigella	13.42
Pseudomonas	2.80
Sutterella	1.00
Herbaspirillum	0.01
Stenotrophomonas	0.01
Actinobacteriota	**30.42**
Bifidobacterium	29.16
Actinomyces	0.74
Collinsella	0.38
Rothia	0.08
Other Actinobacteriota	0.04
Eggerthella	0.02
Firmicutes	**24.02**
Clostridium sensu stricto 1	9.52
Other Firmicutes	5.26
Streptococcus	4.70
Erysipelatoclostridium	2.10
Hungatella	1.30
Enterococcus	1.14
Bacteroidota	**10.02**
Bacteroides	8.97
Parabacteroides	0.67
Prevotella	0.30
Other Bacteroidota	0.04
Grupo *dgA-11*	0.02
Alistipes	0.02
Others	**0.04**
Other taxa	0.04
**Total**	**100**
